# Data on child complementary feeding practices, nutrient intake and stunting in Musanze District, Rwanda

**DOI:** 10.1016/j.dib.2018.09.084

**Published:** 2018-10-03

**Authors:** Vestine Uwiringiyimana, Marga C. Ocké, Sherif Amer, Antonie Veldkamp

**Affiliations:** a Faculty of Geo-Information Science and Earth Observation (ITC), University of Twente, P.O. Box 217, 7500 AE Enschede, The Netherlands; b Department of Food Science and Technology, College of Agriculture Animal Science and Veterinary Medicine, University of Rwanda, PO. Box 3900, Kigali, Rwanda; c National Institute for Public Health and the Environment (RIVM), P.O. Box 1, 3720 BA Bilthoven, The Netherlands

**Keywords:** Complementary feeding practices, Stunting, Nutrient intake, Children, Musanze, Rwanda

## Abstract

Stunting prevalence in Rwanda is still a major public health issue, and data on stunting is needed to plan relevant interventions. This data, collected in 2015, presents complementary feeding practices, nutrient intake and its association with stunting in infants and young children in Musanze District in Rwanda. A household questionnaire and a 24-h recall questionnaire were used to collect the data. In total 145 children aged 5–30 months participated in the study together with their caregivers. The anthropometric status of children was calculated using WHO Anthro software [1] according to the WHO growth standards [2]. The complementary feeding practices together with households’ characteristics are reported per child stunting status. The nutrient intake and food group consumption are presented per age group of children. Also, the percentage contribution of each food groups to energy and nutrient intake in children is reported. The data also shows the association between zinc intake and age groups of children. Using multiple linear regression, a sensitivity analysis was done with height-for-age z-score as the dependent variable and exclusive breastfeeding, deworming table use, BMI of caregiver, dietary zinc intake as independent variables. The original linear regression model and a detailed methodology and analyses conducted are presented in Uwiringiyimana et al. [3].

**Specifications table**

**Table:** 

Subject area	Nutrition
More specific subject area	Nutritional status and complementary feeding practices
Type of data	Table and figure
How data was acquired	Household questionnaire, 24-hour recall questionnaire and anthropometric measurement
Data format	Analysed
Experimental factors	Survey respondents were mothers of young children aged 5–30 months
Experimental features	Anthropometric status of children and their caregivers were collected and analysed using WHO Anthro software. Data processing of nutrient intake was done in Excel 2010 and statistical analysis was conducted using SPSS software version 24.
Data source location	Musanze District, Rwanda
Data accessibility	Data is with this article

**Value of the data**

The data is important for any program or intervention designed to alleviate stunting in children in Rwanda.

• This data is useful to researchers looking for locally conducted research on stunting in children in Rwanda.
• This data is important for complementary feeding practices and stunting in children.
• The food group consumption data can be used for further research on the dietary intake of infants and young children.
• Programs or interventions aiming at improving the diet quality of children focusing on specific nutrients such as micronutrients can use our data as a benchmark of the quality of complementary foods that children consume.
• Our data is useful to inform government, local and international partners working to alleviate stunting in the African region.

## 1 Data

**Fig. 1:**
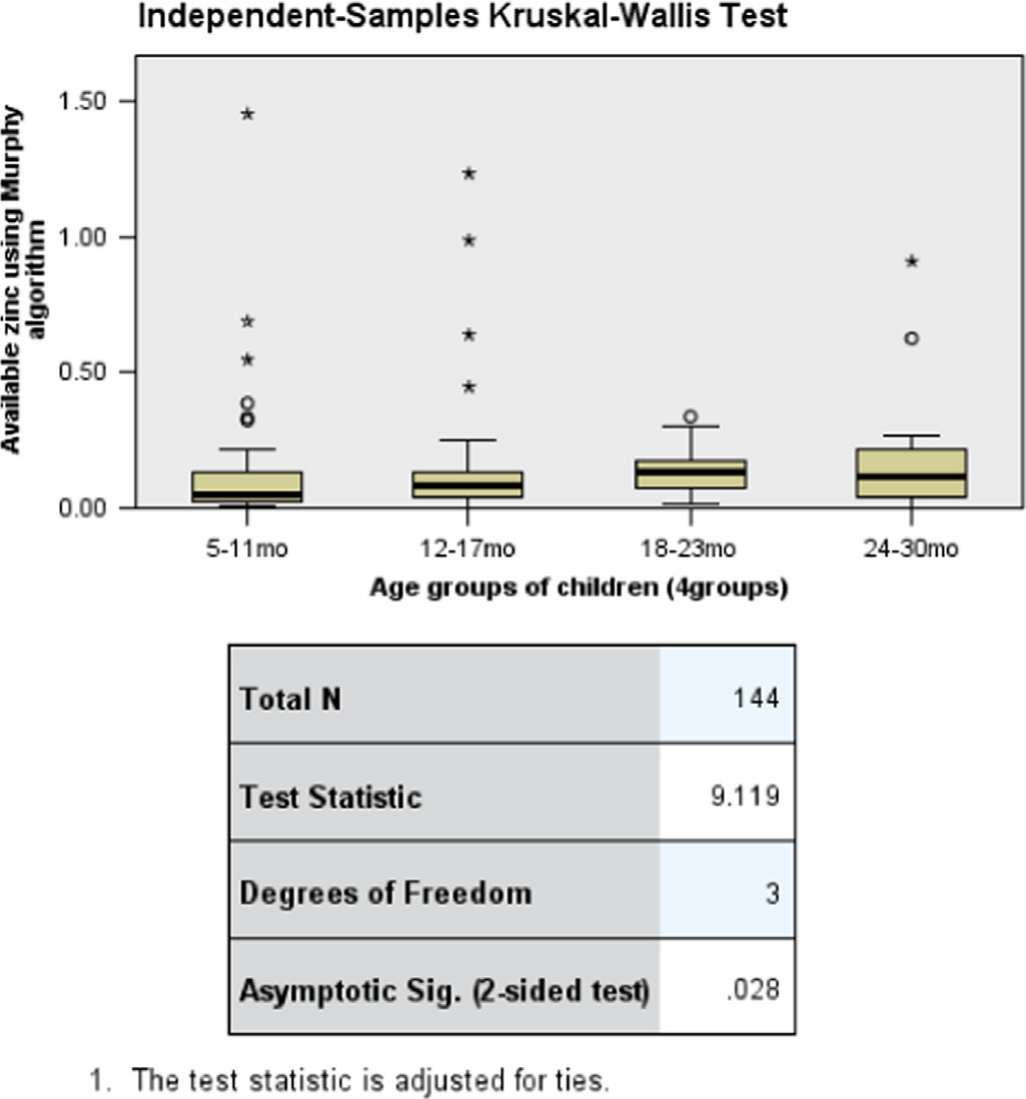
Association between zinc intake and age groups: Independent samples test view for Kruskal-Wallis Test.

**Fig. 2:**
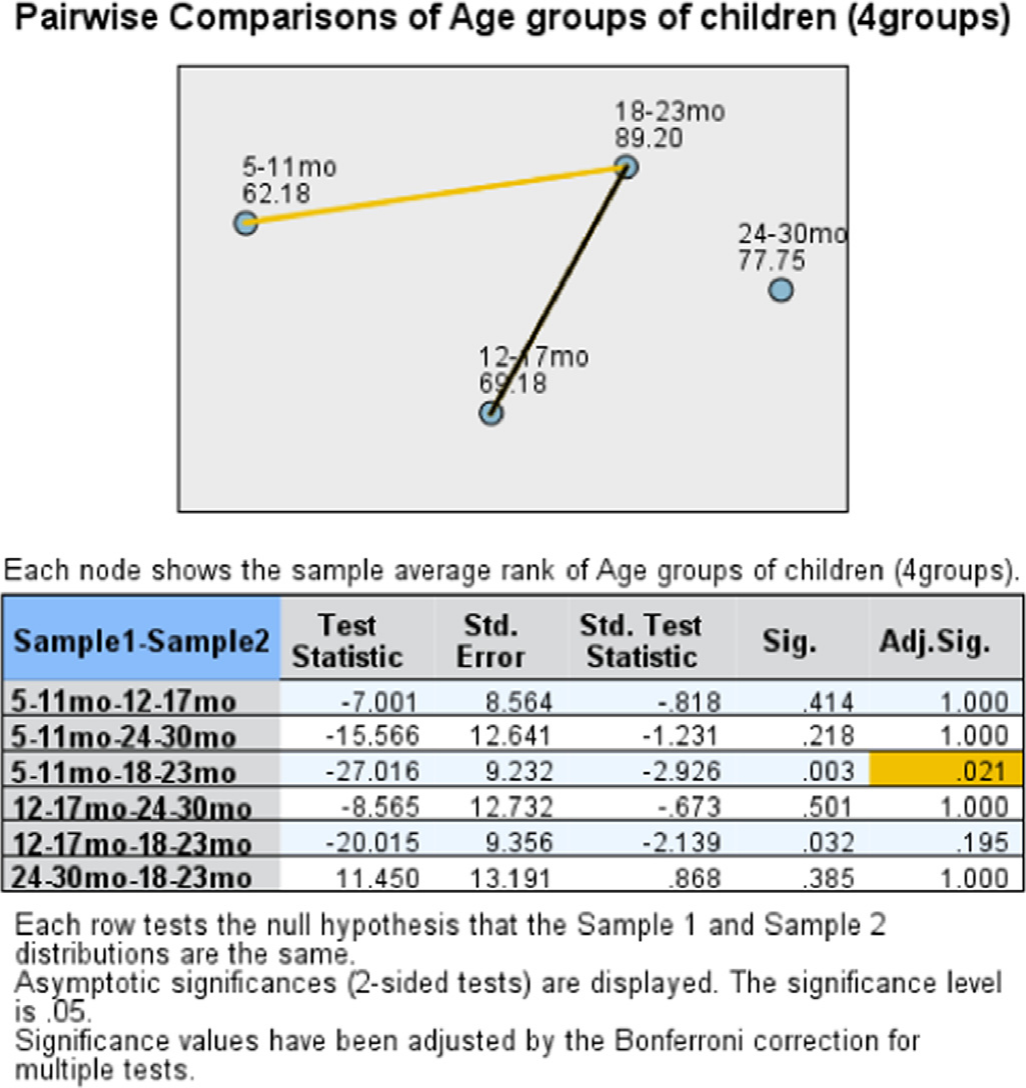
Association between zinc intake and age groups: Pairwise comparisons for Kruskal-Wallis Test.

**Fig. 3:**
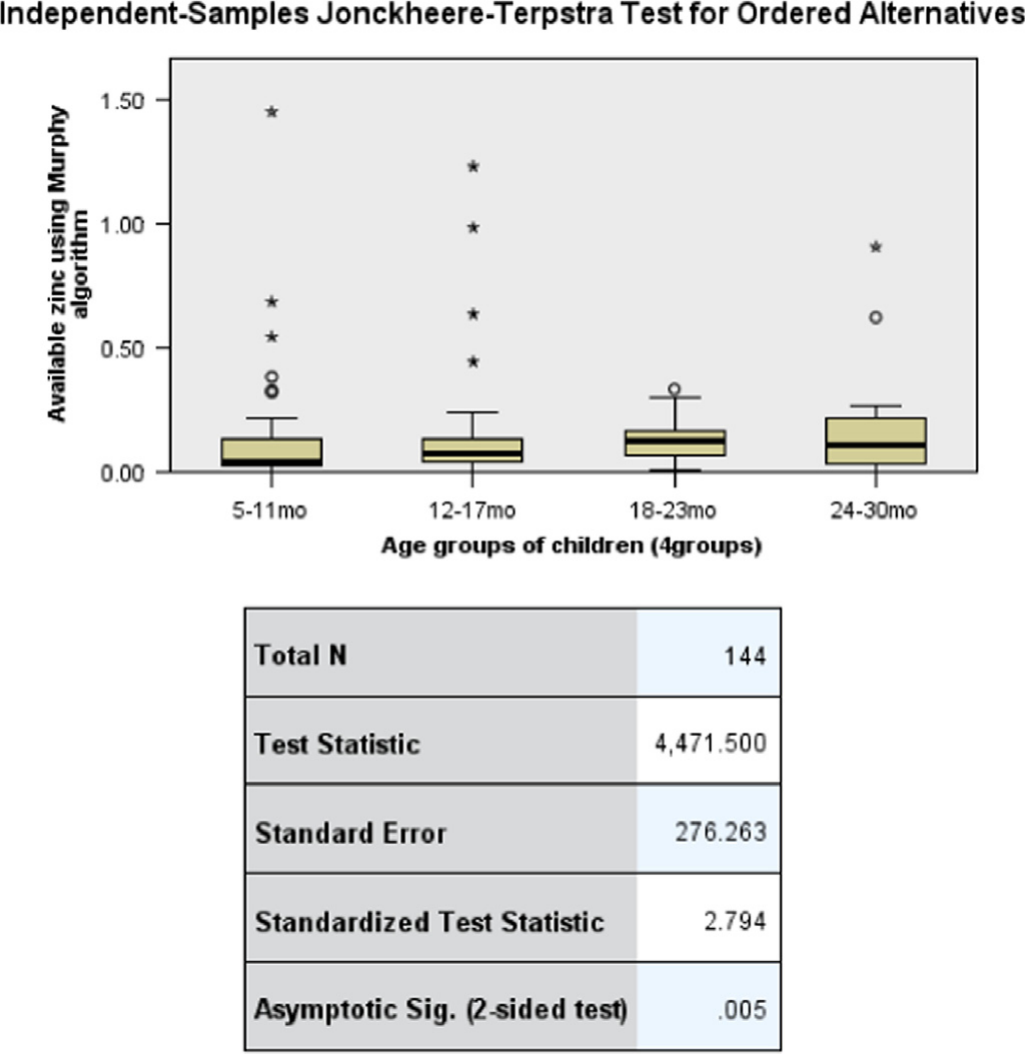
Association between zinc intake and age groups: Independent samples test view for Jonchheere׳s Test for Ordered Alternatives.

The data presents the child complementary feeding practices, nutrient intake and stunting status of children in Musanze District. Table 1 presents the anthropometric status of children namely the stunting, wasting and undernutrition status. Table 2 shows the comparison of stunting, wasting and undernutrition in the District of Musanze and the national prevalence of stunting, wasting and undernutrition reported in the 2015 Demographic and Health Survey. Table 3 shows the complementary feeding practices and household characteristics per stunting status. Tables 4 and 5 portrays the per cent contribution of food groups to energy and nutrient intake; specifically, Table 5 includes the micronutrient powder among the food groups. Table 6 shows the consumption of food groups per age groups in the same children population. Table 7 displays the association between dietary zinc intake and age groups of children using Kruskal-Wallis Test and Jonchheere-Terpstra Test. Fig. 1, Fig. 2, Fig. 3 are derived from Table 7 and display the independent samples test view and pairwise comparisons. Lastly, Table 8 is about the sensitivity analysis model conducted by considering children whose caregivers indicated that the food the child ate the previous day was similar to the child׳s usual intake.

**Table 1:** Nutritional status of children between 5 to 30 months (n = 138) in Musanze District, Rwanda.

Anthropometric status^a^	Frequency (N)	Percentage (%)
Stunting (HAZ <-2)		44
Moderately stunting	38	62
Severe stunting	23	38
Wasting (WHZ <-2)		7
Moderately wasting	6	61
Severe wasting	4	39
Underweight (WAZ <-2)		16
Moderate underweight	18	78
Severe underweight	5	22

a The percentage (%) for moderate and severe categories are given within the respective group of stunting, wasting and underweight.

**Table 2:** Anthropometric status of children aged 5–30 months (n = 138) in Musanze District compared to national prevalence of under 5.

Indicator	Prevalence (Musanze)	National prevalence^a^
Stunting	44	38
Underweight	16	9
Wasting	7	2

a Rwanda Demographic and Health Survey 2015–16 [5].

**Table 3:** Complementary feeding practices and household characteristics of children between 5 and 30 months in Musanze District, Rwanda.

Characteristic	Non-stunted (n = 77)	Stunted (n = 61)	Total (n = 138)	p-value*
	N (%)			
**Complementary feeding practices**				
Pre-weaning food				
Plain water	2 (7)	10 (24)	12 (18)	–
Cow milk	2 (8)	2 (5)	4 (6)	
Traditional herbal mixture	7 (27)	13 (31)	20 (29)	
Fruit juice	6 (23)	10 (24)	16 (24)	
Porridge	7 (27)	4 (9)	11 (16)	
Other	2 (8)	3 (7)	5 (7)	
Reason for pre-weaning				
Inadequate breast milk	3 (12)	3 (7)	6 (9)	–
Sickness of child	7 (27)	11 (26)	18 (26)	
Colic disease	4 (15)	8 (19)	12 (18)	
Child wanted to eat	10 (38)	13 (31)	23 (34)	
Other	2 (8)	7 (17)	9 (13)	
Weaning age groups				–
<6 months	0 (0)	1 (25)	1 (9)	
7–12 months	3 (43)	1 (25)	4 (36)	
13–24 months	4 (57)	2 (50)	6 (55)	
Person responsible for feeding the child				0.022^b^
Respondent	75 (99)	54 (86)	129 (94)	
Other	1 (1)	7 (12)	8 (6)	
Usual food consumed				
Yes	61 (81)	57 (93)	118 (87)	0.038
No	14 (19)	4 (7)	18 (13)	

**Household characteristics**				
Ownership of agricultural land				0.644
Self-owned	31 (56)	27 (61)	58 (59)	
Hired	17 (31)	10 (23)	27 (27)	
Self-owned & hired	7 (13)	7 (16)	14 (14)	
Income generating activity				0.690
None	5 (7)	6 (10)	11 (8)	
Commerce	8 (10)	6 (10)	14 (10)	
Agriculture	40 (52)	25 (41)	65 (48)	
Domestic work	18 (24)	19 (31)	37 (27)	
Employment (formal &informal)	5 (7)	5 (8)	10 (7)	
Water source for household				–
Piped water	58 (76)	43 (70)	101 (73)	
Water from spring	4 (5)	7 (12)	11 (8)	
Rainwater	2 (3)	3 (5)	5 (4)	
Surface water (river /dam/ stream)	12 (16)	8 (13)	20 (15)	
Water treatment in the household				
Nothing	38 (51)	34 (56)	72 (53)	–
Boil	26 (35)	19 (31)	45 (33)	
Add bleach/chlorine	7 (9)	6 (10)	13 (10)	
Other	4 (5)	2 (3)	6 (4)	
Time taken to/from water collection point				0.181
Less than 30 min	49 (64)	32 (53)	81 (59)	
Between 30–60 min	19 (25)	16 (26)	35 (26)	
More than 1 h	8 (11)	13 (21)	21 (15)	
Biofortified crops grown by household				0.445 b
Yes	0 (0)	1 (2)	1 (1)	
No	76 (100)	60 (98)	136 (99)	
Improved seeds use by household				0.754 b
Yes	7 (9)	4 (7)	11 (8)	
No	69 (91)	57 (93)	126 (92)	
Industrial fertilizers use by household				0.801
Yes	47 (62)	39 (64)	86 (63)	
No	29 (38)	22 (36)	51 (37)	

* *p*-value: two-sided, obtained through Pearson Chi-square.

b Exact Sig. (2-sided) from Fisher׳s Exact Test. - If n was too low for statistical testing.

**Table 4:** Percent contribution of food groups to energy and nutrient intake from complementary feeding of children (aged 5–30 months) from Musanze District^a^.

Food groups	Energy	Protein	Fat	Carbohydrate	Iron	Calcium	Magnesium	Zinc	Phytates	Vitamin A	Vitamin C
Cereals	35	45	13	58	22	14	49	32	52	0	0
Roots and tubers	4	3	1	8	2	2	4	3	4	0	9
Legumes	3	10	0	5	6	6	8	7	20	2	4
Nuts, seeds and their products	5	10	6	2	3	1	10	6	22	0	0
Milk and milk products	1	1	0	1	0	3	1	1	0	0	0
Meat, poultry, fish	3	15	3	0	3	23	5	17	0	0	0
Egg or egg products	1	5	1	0	1	1	0	2	0	1	0
Fruits and fruit juices	4	2	1	7	2	1	6	2	0	1	22
Vegetables, herbs and vegetable products	5	10	1	8	60	48	18	15	1	18	64
Fats and oils	36	0	72	0	0	0	0	15	0	77	0
Sugar and sweets	5	0	0	12	1	0	0	0	0	0	0

a Micronutrient powder (MNP) was not included

**Table 5:** Percentage contribution of food groups to energy and nutrient intake from complementary feeding with micronutrient powder (MNP) included^a^.

Food groups	Energy	Protein	Fat	Carbohydrate	Iron	Calcium	Magnesium	Zinc	Phytates	Vitamin A	Vitamin C
Cereals	34	45	13	55	5	14	49	6	52	0	0
Roots and tubers	4	3	1	7	0	2	4	0	4	0	2
Legumes	3	10	0	5	1	6	8	1	20	1	1
Nuts, seeds and their products	5	10	6	1	1	1	10	1	22	0	0
Milk and milk products	1	1	0	1	0	3	1	0	0	0	0
Meat, poultry, fish	3	15	3	0	1	23	5	3	0	0	0
Egg or egg products	1	5	1	0	0	1	0	0	0	1	0
Fruits and fruit juices	4	2	1	6	0	1	6	0	0	0	6
Vegetables, herbs and vegetable products	5	10	1	8	12	48	18	3	1	7	17
Fats and oils	36	0	72	0	0	0	0	3	0	28	0
Sugar and sweets	5	0	0	11	0	0	0	0	0	0	0
Other (MNP)	0	0	0	4	80	0	0	82	0	63	74

a Micronutrient powder had been used by only 38% of caregivers in the last four weeks that preceded the survey. No caregiver had used micronutrient powder in their child׳s diet the day that preceded the survey.

**Table 6:** Prevalence of food group consumption per age groups reported in a single 24-h recall in children aged 5–30 months from Musanze District.

Food groups		5–11mo (n=49)	12–17mo (n=46)	18–23mo (n=35)	24–30mo (n=14)	Total (n=144)
		N (%)				
Grain, roots & tubers	No	1 (1)	3 (2)	0 (0)	1 (1)	5 (3)
	Yes	48 (33)	43 (30)	35 (24)	13 (9)	139 (97)
Legumes & nuts	No	8 (6)	11 (8)	8 (6)	4(3)	31 (22)
	Yes	41 (28)	35 (24)	27 (19)	10 (7)	113 (78)
Dairy products (milk, yogurt, cheese)	No	46 (32)	46 (32)	35 (24)	14 (10)	141 (98)
	Yes	3 (2)	0 (0)	0 (0)	0 (0)	3 (2)
Flesh foods (meat, fish, poultry & liver/organ meats)	No	44 (31)	41 (28)	35 (24)	13 (9)	133 (92)
	Yes	5 (3)	5 (3)	0 (0)	1 (1)	11 (8)
Eggs	No	49 (34)	46 (32)	32 (22)	14 (10)	141 (98)
	Yes	0 (0)	0 (0)	3 (2)	0 (0)	3 (2)
Vitamin A rich fruits & vegetables	No	11 (8)	11 (8)	9 (6)	5 (3)	36 (25)
	Yes	38 (26)	35 (24)	26 (18)	9 (6)	108 (75)
Other fruits & vegetables	No	22 (15)	24 (17)	23 (16)	10 (7)	79 (55)
	Yes	27 (19)	22 (15)	12 (8)	4 (3)	65 (45)

**Table 7:** Association between zinc intake and age groups (Kruskal-Wallis test).

**Hypothesis Test Summary**				
	**Null Hypothesis**	**Test**	**Sig.**	**Decision**
**1**	The distribution of Available zinc using Murphy algorithm is the same across categories of Age groups.	Independent-Samples Kruskal-Wallis Test	.028	Reject the null hypothesis.
**2**	The distribution of Available zinc using Murphy algorithm is the same across categories of Age groups.	Independent-Samples Jonckheere-Terpstra Test for Ordered Alternatives	.005	Reject the null hypothesis.

Asymptotic significances are displayed. The significance level is .05.

**Table 8:** Sensitivity analysis model of predictors of height-for-age z-scores in children aged 5–30 months in Musanze District, Rwanda^a^.

Variables	β	*p*-value	95% CI for β	
			Lower bound	Upper bound
Age (months)				
Age group 12–17mo vs 5–11mo	−0.92	0.000	−7.55	−3.10
Age group 18–23mo vs 5–11mo	−2.19	0.073	−1.94	0.09
Age group 24–30mo vs 5–11mo	−2.83	0.000	−3.13	−1.25
Exclusive breastfeeding (yes)	0.79	0.001	−4.43	−1.23
Use of deworming tablets (yes)	1.93	0.005	0.23	1.35
BMI of caregiver (kg/m^2^)	0.12	0.006	0.03	0.21
Dietary zinc intake (mg)	1.13	0.178	−0.52	2.79
Interaction terms between age groups and energy intake				
Age group 12–17mo*energy intake	−0.002	0.080	−0.004	0.000
Age group 24–30mo*energy intake	−.002	0.175	−0.005	0.001

a The sensitivity analysis model was limited to 116 children whose intake on the recalled day was similar to their usual intake. β: Regression coefficient. CI, confidence interval.

## 2 Experimental design, materials and methods

The data presented was obtained through a cross-sectional survey conducted in the district of Musanze. A detailed methodology is given elsewhere [3]. Ethical approval to collect the data was obtained through the Institutional Review Board of the College of Medicine and Health Sciences in Rwanda. An informed consent was obtained from all participating caregivers. A household questionnaire was used to collect information on socioeconomic status, complementary feeding practices, health and anthropometric status of children. An interactive and multi-pass 24-h recall questionnaire, adapted and validated for use in developing countries [4], was used to collect information on dietary intake. A total of 145 children participated in the study. A single 24-h recall with the caregiver as the respondent was conducted. Information on usual intake of children was also collected.

There was a statistically significant difference in zinc intake between age groups, *H* (3) = 9.12, *p* = 0.028. Pairwise comparisons with adjusted *p*-values showed that there was a significant difference in zinc intake between the age group of 5–11 months and 18–23 months (*p* = 0.021). On the other hand, there was no significant difference in zinc intake between age group of 5–11 months compared to the age group of 12–17 months (*p* = 1.00) and 24–30 months (*p* = 1.00). There were also no significant differences in zinc intake between the age group of 12–17 months and the age groups of 24–30 months (*p* = 1.00) and age group of 18–23 months (*p* = 0.195). Finally, there were no significant differences in zinc intake between the age groups of 24–30 months and the age group of 18–23 months (*p* = 1.00).

The Jonchheere-Terpstra׳s test revealed a significant trend in the data: as the age of children increased, zinc intake increased, *J* = 4471, *z* = 2.794, *p* = 0.005.

## References

[1] WHO (2011). WHO Anthro for Personal Computers, Version 3.2.2: Software for Assessing Growth and Development of the World׳s Children.

[2] WHO (2006). WHO Child Growth Standards: Length/Height-for-Age, Weight-for-Age, Weight-for-Length, Weight-for-Height and Body Mass Index-for-Age: methods and Development.

[3] Uwiringiyimana V., Ocké M.C., Amer S., Veldkamp A. (2018). Predictors of stunting with particular focus on complementary feeding practices: a cross-sectional study in the Northern Province of Rwanda. Nutrition.

[4] Gibson R.S., Ferguson E.L. (2008). An Interactive 24-Hour Recall for Assessing the Adequacy of Iron and Zinc Intakes in Developing Countries.

[5] NISR, MOH, ICF International (2015). Rwanda Demographic and Health Survey 2014–15.

